# Supplementary material for: Molecular Mechanisms of RSV and Air Pollution Interaction: A Scoping Review
Source: Int J Mol Sci. 2022 Oct 21;23(20):12704. doi: 10.3390/ijms232012704 (PMC9604398; doi:10.3390/ijms232012704)
Supplement: Supplementary file 1 [file ijms-23-12704-s001.zip › S1. PubMed search strategy.pdf]

PubMed search strategy:

Search within categories was executed with the use of OR, while all the major categories were connected with AND. The PubMed translate engine was used.

Major categories:

A. Etiology
B. Air pollution
C. Molecular mechanism

MeSH terms relating to the etiology:

1. Respiratory syncytial virus.

Keywords terms relating to the pathogen:

MeSH terms relating to the air pollution:

1. Air pollution
2. Environmental pollutants
3. Air pollutants
4. Particulate matter
5. Carbon monoxide
6. Nitrogen dioxide
7. Sulfur dioxide

Keywords terms relating to the air pollution:

1. Ambient pollution
2. PM2.5
3. PM 2.5
4. PM10
5. PM 10
6. Carbon oxide

MeSH terms relating to the pathomechanism:

1. Pathology, molecular
2. Up-regulation
3. Down-regulation

Keywords terms relating to the pathomechanism:

1. Pathomechanism
2. Regulat*

regulat* OR susceptib* OR increas* OR decreas* OR induc* OR chang* OR pathogenesis OR supress* OR interacti* OR enhanc* OR block* OR respon* OR dysregulat* OR receptor OR epithel* OR surface OR alter*
