# Supplementary material for: Molecular Mechanisms of RSV and Air Pollution Interaction: A Scoping Review
Source: Int J Mol Sci. 2022 Oct 21;23(20):12704. doi: 10.3390/ijms232012704 (PMC9604398; doi:10.3390/ijms232012704)
Supplement: Supplementary file 1 [file ijms-23-12704-s001.zip › Table S2. The characteristics of the studies included in the scoping review..pdf]

Author	Air pollutant	Material/subjects	Country	Mechanism	Main results
Becker and Soukup 1999 [84]	NO_2 (different concentrations: 0.5, 1.0, and 1.5 ppm)	Human bronchial epithelial cell line BEAS-2B	USA	Viral replication IL- 6, IL-8	RSV internalization increased by 0.5 ppm exposure and decreased by 1.5 ppm exposure; The release of infectious virus 48 h postexposure was reduced only after 1.5 ppm NO_2 exposure; Reduced IL-6, IL-8
Becker, Soukup 1999 "Exposure to urban air particulates alters…" [67]	PM_{10} (EHC-93)	human bronchial epithelial cell line BEAS-2B subclone S6 and human alveolar macrophage from BAL from volunteers	USA	Viral uptake IL-8, MIP-1alpha, MIP-1beta, MCP-1, RANTES	50% decrease in viral uptake of virus by AM; MCP-1 production inhibited; MIP-1 and IL-8 levels lower than expected from a combined response;

					RSV-induced production of RANTES by epithelial cells was decreased in the presence of AM, but not affected by PM10
Castro 2008 [91]	Cigarette smoke condensate	A549, human alveolar type II–like epithelial cells, and 293, a human embryonic kidney epithelial cell line	USA	IL-8 (gene and protein expression), MCP-1 (gene and protein expression), nuclear factor kappa B (NF-kB)	Increased IL-8 and MCP-1 gene and protein expression Enhanced activation of interferon regulatory factor (IRF)-1 and IRF-7 Enhanced activation of NF-kB, and increased NF-kB–driven gene transcription. Increased chemokine expression in airway epithelial cells

Castro 2011 [72]	cigarette smoke extract	Human plasmacytoid dendritic cells	USA	IFN-α<br>IL-1β, IL-6, IL-10, TNF-α, CCL2 (MCP-1), CCL3 (MIP-1a), CCL5 (RANTES), CXCL10 (IP-10), CXCL8 (IL-8)<br><br>qRT-PCR for IRF-7, TLR7, TLR9 and 18S<br>Intracellular staining of IRF-7	Inhibited RSV-induced IFN-α in pDC, inhibited release of IL-1β, IL-10 and CXCL10.<br>Not altered: IL-6, TNF-α, CCL2, CCL3, CCL5 and CXCL8<br>Decreased the expression of toll-like receptor (TLR)-7 and interferon regulatory factor (IRF)-7 in RSV-infected pDC.<br>CSE prevented IRF-7 activation.
Chakraborty 2017 [42]	TiO2-NP	Human primary bronchial epithelial cells)	USA	Expression of NGF and its receptors: TrkA and p75NTR<br>Autophagy (beclin-1 expression)<br>Cell death (FACS after annexin V/propidium iodide staining)	RSV infection efficiency more than doubled;<br>NGF and its TrkA receptor upregulated;<br>Silencing NGF gene expression with siRNA significantly

					inhibited RSV infection; Increase in necrotic cell death and reduction in apoptosis; Increase in expression of the autophagosomal gene beclin-1; Pharmacological inhibition of beclin-1-> increased apoptotic rate and lower viral load
Cruz-Sanchez 2013 [43]	Mimics of ambient particulate matter (PM_{10})	human epithelial-2 (HEp-2) cells 1HAEo-cells- normal human airway epithelial cells transformed with Simian virus 40	Canada	IL-6, IL-8 RSV survival	Increased IL-6 and IL-8 Accelerated viral entry (the mechanism involved endocytic pathway; dynamin dependent)
Foronjy 2014 [86]	Cigarette smoke	C57BL/6J mice (repeated RSV	USA	Histological analysis	Enhanced influx of macrophages,

		exposition- 6 times)		Protease and Cytokine Measurements Intracellular Signaling (cathepsin E, S, G, K, W, Z, MMP-28, actin) Terminal Deoxynucleotidyl Transferase dUTP Nick Endlabeling (TUNEL) Analysis Viral titers Phosphatase Levels (PP2A and PTP1B)	neutrophils and lymphocytes; Most pronounced around the vasculature and bronchial airways; Airspace enlargement and fibrosis; Induced cytokine (IL-1α, IL-17, IFN-γ, KC, IL-13, CXCL9, RANTES, MIF and GM-CSF) and protease (MMP-2, -8, -12, -13, -16 and cathepsins E, S, W and Z) expression.; Enhanced apoptosis; Reduced protein phosphatase 2A (PP2A) and protein tyrosine phosphates

					(PTP1B) expression and activity
Foronjy 2016 [87]	Cigarette smoke	lung BALF from age-matched healthy control subjects, smokers, and subjects with COPD<br><br>Ptp1b (Ptpn1 gene) knockout (-/-) mice<br><br>human primary small airway epithelial and mouse bone marrow derived macrophages	USA	Protein tyrosine phosphatase 1B (PTP1B)<br><br>S100A9<br><br>Caspase-3, p-IκBα, IκBα (Ser32), p-p38 (Thr180/Tyr182), p38, TRIF, MyD88, IRAK1, pp44/42 MAPK (Erk1/2) (Thr202/Tyr204), ERK1/2, β-actin<br><br>Viral titer	Ptp1b -/- mice -> exaggerated immune cell infiltration, damaged epithelial cell barriers, cytokine production, increased apoptosis, and elevated expression of S100A9<br>Pre-exposure to cigarette smoke desensitized PTP1B activity -> enhanced S100A9 secretion and inflammation.<br>S100A9 levels in human BALF- an inverse relationship with lung function in healthy subjects,

					smokers and COPD subjects. HBEC from COPD donors-secreted more S100A9 following RSV infection.
Groskreutz 2009 [44]	Cigarette smoke extract	Primary human tracheobronchial epithelial	USA	Cytokine measurements<br>Cell Death Detection (ELISA + TUNEL Assay)<br>Cell Survival Assays<br>Fluorescent RSV/DAPI Staining<br>Plaque Assay<br>Western blot detection of cleaved caspases 3 and 7 showed	Less apoptosis;<br>Necrosis rather than apoptosis;<br>Less live cells, but increased viral load;<br>The effect inhibited by pretreatment with N-acetylcysteine and aldehyde dehydrogenase;<br>Cigarette Smoke Prevents RSV- and Staurosporine-Induced Caspase 3 and 7 Activation;<br>p53 or XIAP not altered

Harrod 2003 [29]	Diesel engine emissions	C57Bl/6 mice	USA	Inflammatory cells in BALF<br><br>Lung histology<br><br>TNF-α, IFN-γ<br><br>Clara cell secretory protein, CCSP (=CC-10 or CC-16)<br><br>Surfactant protein A (SP-A) and proprotein B (proSP-B)	Increased viral gene expression; Increased cell numbers in BALF, domination of alveolar macrophages (AM, >95%); Augmented lung inflammation (peribronchial and peribronchiolar regions) and airway epithelial remodeling (loss of the normal cuboidal appearance of nonciliated bronchiolar epithelial (Clara) cells; increased appearance of pseudostratified, columnar epithelial cell morphology and apparent airway

					epithelial cell sloughing); a dose-related increase in lung histolopathology; Mucous cell metaplasia; Increased TNF-$\alpha$and IFN-γ; Attenuation of CCSP production, the numbers of CCSP-producing cells diminished; Surfactant proprotein B (proSP-B) decreased; Decreased SP-A
Hashiguchi [85]	TiO2-NP	BALB/c mice	Japan	IL-2, IL-4, IL-10, IFN-γ, RANTES (CCL5), MIP-1α (CCL3) Flow cytometric analysis Histological examination Viral titres	Increased IFN-$\gamma$and RANTES Viral titers unaffected An increase in the infiltration of lymphocytes into the alveolar septa in lung tissues

Hirota 2015 [73]	PM10 (EHC93)	human airway epithelial cell line (HBEC-6KT)	Canada	IL-1β, CCL20, GM-CSF, TSLP, IL-33, and IL-25<br>Differential Interference Contrast and Scanning Confocal Microscopy	No effect on IL-1β responses
Hobson and Everard 2007 [48]	Nitric oxide	human monocyte-derived dendritic cells (DCs)	UK	Viral titres<br>Viral persistence	RSV replication outside the RSV epidemic season after exposure to nitric oxide
Ivanciuc 2019 [112]	side-stream tobacco smoke	cystathionine γ-lyase enzyme (CSE)- deficient and wild-type mice	USA	Aairway hyperresponsiveness (AHR) to methacholine challenge<br>Viral replication<br>Inflammatory cytokines and chemokines	CSE-deficient mice: more severe clinical disease, airway obstruction and AHR, enhanced viral replication and lung inflammation, a significant increase in the number of neutrophils in BALF, increased

					levels of inflammatory cytokines and chemokines.
Kaan and Hegele 2003 [47]	PM10 (EHC-93)	Guinea pig alveolar macrophages	Canada	IL-6, IL-8<br>RSV Yield (the amount of viral replication per RSV-immunopositive cell)	Enhanced secretion of TNF-α, decreased RSV Yield
Lambert 2003 "Effect of Preexposure to Ultrafine Carbon Black…" [79]	Preexposure to ultrafine carbon black	BALB/c mice	USA	TNF-α<br>IL-13<br>IFN-γ-inducible protein 10 (IP-10) mRNA<br><br>lymphotactin, RANTES, eotaxin, MIP-1α, MIP-1β, MIP-2, MCP-1, and TCA-3, housekeeping genes L32 and GAPDH<br><br>RNA templates for interleukins 10, 13, 15, 9, 2, 6, and IFN-$\gamma$and interleukins 10, 1α, 1β, 1Rα, 6,	Reduced TNF-αin BALF on days 1 and 2 of infection; a reduction in BALF lymphocyte numbers on day 4, reduced IP-10, lymphotactin, and IFN-γ mRNAs;<br>No changes in RANTES, eotaxin, MCP-1, MIP-1;<br>Lower viral titers on days 2– 4 of infection; by day

				and IFN-γ, L32 and GAPDH genes	7 neutrophil numbers, proinflammatory cytokine mRNA expression, and protein levels of TNF-$\alpha$and IL-13 increased
Lambert 2003 "Ultrafine Carbon Black Particles Enhance…"[96]	Ultrafine carbon black after RSV infection	BALB/c mice	USA	AHR to methacholine<br><br>bronchoalveolar lavage and cell differentials<br><br>MIP-1α, MCP-1, and RANTES<br><br>RSV antigen quantitation<br><br>Laser capture microdissection of alveolar epithelium<br><br>Reverse transcription (MIP-	Viral titer and clearance unaffected by CB exposure; Neutrophil numbers elevated on days 4 and 7, and lymphocyte numbers were higher on days 4 and 14 of infection in CB-exposed, RSV-infected mice; Enhanced AHR, BAL total protein, MCP-1,

				1α and the housekeeping gene L32)	MIP1-α, RANTES; MIP-1-α mRNA expression increased in the alveolar epithelium, where ultrafine particles deposit in the lung
Mebratu 2016 [78]	Cigarette smoke	C57BL/6 mice	USA	Lung inflammatory cells, histology and morphometry<br><br>TUNEL assay<br><br>mRNA levels for IL-17a, IL-17c, IL-17d, IL-17 f. IL-1β, IL-12b, IL-18, IL-23a, Ccl-2, Ccl-7 at day 4 post infection in lung tissue<br><br>Quantitative RT-PCR for MMP-12 mRNA at day 14 post infection.<br><br>Viral load	Inflammation characterized y by lymphocytes and neutrophils; Robust emphysema with enhanced MMP-12 mRNA expression and TUNEL positivity; Increased the levels of IL-17 (a, b, d, f), IL-1β, IL-12b, IL-18, IL-23a, Ccl-2, Ccl-7 mRNAs in the lungs; By day 4 lower copy number of

					viruses, but higher on day 14.
Modestou 2010 [45]	Cigarette smoke extract	Human trachea and bronchial samples<br><br>Primary human tracheobronchial epithelial cells	USA	Antibodies against:<br>- interferon regulatory factor-9 (IRF-9)<br>- ICAM-1<br>- Stat1<br>- tyrosine-701 phosphorylated human Stat1<br>- heat shock protein (HSP)-90<br>- β-actin<br>- serine-727 phosphorylated human Stat1<br>- serine-727 phosphorylated human Stat1<br>ICAM-1<br>Epithelial Cytotoxicity Assays<br>Glutathione Assay	Inhibited IFN-γ-dependent gene expression (not due to cell loss or cytotoxicity);<br>Inhibited IFN-γ-induced Stat1 phosphorylation;<br>Decreased inhibitory effect of IFN-γ on RSV mRNA and protein expression;<br>CSE effects on IFN-γ-induced Stat1 activation, antiviral protein expression, and inhibition of RSV infection decreased by glutathione augmentation of epithelial cells (N-ACC or glutathione monoethyl ester)

Phaybouth 2006 [46]	side-stream cigarette smoke	Newborn BALB/cmice (RSV infection twice)	USA	Pathologic analysis<br>RT-PCR analysis of viral gene expression<br>IL-5, IL-12, IL-13, and IFN-γ<br>CCSP	Neutrophilia and decreased CCSP levels in response to RSV rechallenge;<br>An increase in BALF eosinophils, reduced levels of IFN-γ, and IL-12, decreased lung tissue inflammation, and decreased mucus production after RSV rechallenge;<br>Increased viral gene expression
Poon 2019 [111]	Cigarette smoke- mice<br><br>COPD patients<br>Smokers	Mice exposed to cigarette smoke<br><br>BALF from healthy never smokers, smokers, and COPD patients	USA	LIF<br>LIFR	LIF in human BALF reduced in both smokers and COPD patients;<br>HBE cells isolated from COPD patients produced less LIF during RSV infection;

		Human bronchial epithelial (HBE)			Reduced LIF and its receptor (LIFR) in mice lungs Smoke-exposed animals had reduced LIF expression during RSV infection.
Smallcombe 2020 [37]	Titanium dioxide nanoparticles	Immortalized human bronchial epithelial cells<br><br>C57BL/6 mice	USA	Epithelial cell barrier integrity<br>Viral titer<br>Reactive oxygen species (ROS) generation	Apical junctional complex (AJC) disruption induced by RSV amplified by TiO_2-NP;<br>Increased viral infection in epithelial cells;<br>TiO_2 induced generation of ROS, and pretreatment with antioxidant (N-ACC) reversed barrier dysfunction;<br>In vivo, RSV-induced injury and AJC disruption were

					augmented in the lungs of mice given TiO2-NP; Airway inflammation was exacerbated (WBC infiltration into the BAL), along with exaggeration of peribronchial inflammation and AJC disruption
Raza 1999 [77]	water-soluble cigarette smoke extract (CSE), nicotine, cotinine	monocytes of the blood from donors	UK	TNF-α<br>nitric oxide (NO)(as nitrites)	Increased TNF-α and decreased NO, higher proportion of extreme responses
Soukup, Koren, Becker 1993 [83]	ozone	Human alveolar macrophages	USA	IL1, IL-6, TNF<br><br>Percentage RSV-infected AM<br><br>Amount of infectious virus released	No difference in percentage of infected AM or in the amount of infectious RSV produced; Effects on IL-1 and Il-6 secretion were only found with the lowest

					levels of infection which induced cytokine release;<br>TNF production unaffected by ozone
Urrego 2009 [98]	Nicotine exposure	Rats	USA	RT-PCR for NGF, brain-derived neurotrophic factor (BDNF), p75NTR, trkA, trkB, and the housekeeping gene β-actin<br><br>NGF, BDNF, p75NTR, trkA, trkB, protein levels<br><br>Extravasation of albumin from airway blood vessels.	An additive effect on NGF expression, exaggerated neurogenic airway inflammation (abolished by selective inhibition)

Table S2. The characteristics of the studies included in the scoping review.
