# Supplementary material for: Molecular Mechanisms of RSV and Air Pollution Interaction: A Scoping Review
Source: Int J Mol Sci. 2022 Oct 21;23(20):12704. doi: 10.3390/ijms232012704 (PMC9604398; doi:10.3390/ijms232012704)
Supplement: Supplementary file 1 [file ijms-23-12704-s001.zip › Table S3. JBI Checklist for Quasi-Experimental Studies (non-randomized experimental studies).pdf]

**D1**	Is it clear in the study what is the 'cause' and what is the 'effect' (i.e. there is no confusion about which variable comes first)?
**D2**	Were the participants included in any comparisons similar?
**D3**	Were the participants included in any comparisons receiving similar treatment/care, other than the exposure or intervention of interest?
**D4**	Was there a control group?
**D5**	Were there multiple measurements of the outcome both pre and post the intervention/exposure?
**D6**	Was follow up complete and if not, were differences between groups in terms of their follow up adequately described and analyzed?
**D7**	Were the outcomes of participants included in any comparisons measured in the same way?
**D8**	Were outcomes measured in a reliable way?
**D9**	Was appropriate statistical analysis used?

Table S3. JBI Checklist for Quasi-Experimental Studies (non-randomized experimental studies).
