# Supplementary material for: Molecular Mechanisms of RSV and Air Pollution Interaction: A Scoping Review
Source: Int J Mol Sci. 2022 Oct 21;23(20):12704. doi: 10.3390/ijms232012704 (PMC9604398; doi:10.3390/ijms232012704)
Supplement: Supplementary file 1 [file ijms-23-12704-s001.zip › Table S4. Funding sources of the studies included in the review..pdf]

paper	year	country	funding
Becker and Soukup	1999	USA	The research described in this article has been supported by the U.S. Environmental Protection Agency.
Becker and Soukup "Exposure to urban air particulates alters…"	1999	USA	The research described in this article has been supported by the U.S. Environmental Protection Agency.
Castro	2008	USA	National Institute of Environmental Health Science (ES06676); Flight Attendant Medical Research Institute Clinical Investigator Awards to A.C. and R.P.G.; National Institute of Environmental Health Science training grant (T32 ES07254) supported S.M.C.; and National Institutes of General Medical Sciences Ruth L. Kirschstein National Research Service Award (F31 GM072231)
Castro	2011	USA	This work was supported by the Flight Attendant Medical Research Institute (FAMRI) Young Clinical Scientist Award to A. G-P.
Chakraborty	2017	USA	This work was supported in part by the U.S. National Institutes of Health grant RO1- HL61007 to Dr. Giovanni Piedimonte. Image acquisition and data analysis were performed at the WVU Microscope Imaging Core Facility, which was supported in part by the NIH grant P20RR016440. Flow cytometry experiments were performed in the WVU Flow Cytometry Core Facility, which was supported in part by National Institutes of Health grants RR106440 and RR020866. We are indebted to Dr. Mark Peeples (Nationwide Children's Hospital Research Institute, Columbus, OH) and Dr. Peter Collins (National Institutes of Health, Bethesda, MD) for providing the RFP-tagged RSV.
Cruz-Sanchez	2013	Canada	This work was supported by the Canadian Institutes for Health Research/Allergen Grant CIHR79632, the Heart & Stroke Foundation of Canada, an unrestricted grant from Johnson & Johnson Corporate Office of Science and

			Technology, the Natural Sciences and Engineering Research Council of Canada, and Simon Fraser University. The UBC BioImaging facility is funded by the Canadian Foundation of Innovation (CFI), the British Columbia Knowledge Development Fund (BCKDF), the UBC Blusson Fund, and the Natural Sciences and Engineering Research Council of Canada (NSERC). The imaging facility at the James Hogg Research Centre is CFI-funded. The 1HAEo- cells were a kind gift from Dr. D. Gruenert, University of Vermont, Burlington, VT. The GFP-RSV was generously provided by Dr. Mark E. Peeples, Children's Research Institute, Columbus, OH. The Dyn K44 psrα was a kind gift from Dr. Mark Marsh at the MRC Laboratory for Molecular Cell Biology, University College London, U.K. Tillie L. Hackett is a CIHR, Canadian Lung Association (CLA) and GlaxoSmithKline Inc. (GSK), Integrated and Mentored Pulmonary and Cardiovascular Training (IMPACT) and a Michael Smith Foundation for Health Research (MSFHR) postdoctoral fellow. Darryl A. Knight is the Canada Research Chair in Airway Disease and MSFHR Career Investigator and Senior Scholar. Stephan F. van Eeden is a MSFHR Senior Scholar and the GSK/CIHR Professor in Chronic Obstructive Pulmonary Disease.
Foronjy	2014	USA	This work was supported by grants made available to P.G. (Flight Attendant Medical Research Institute (YCSA 113380)) (http://www.famri.org/intro/) and to R.F. (US National Institutes of Health 5R01HL098528-04) (http://www.nih.gov/).
Foronjy	2016	USA	This work was supported by grants made available to P.G. (Flight Attendant Medical Research Institute (YCSA 113380), R.F. (US National Institutes of Health 5R01HL098528-04) and M.S. (Flight Attendant Medical Research Institute 103027). The authors would like to thank the James P. Mara Center for Lung Disease of the Pulmonary Division of Mount Sinai Roosevelt for their support and Dr. Gerard Turino and Dr. Charles Powell.
Groskreutz	2009	USA	This work was supported by a VA Merit Review grant; NIH: HL089392-02, HL073967-01, HL077431-01, HL075559-04, R01 HL079901-01A1,

			AI 063520, and RR00059 from the General Clinical Research Centers Program, NCRR, NIH.
Harrod	2003	USA	The authors wish to thank the National Environmental Respiratory Center (NERC) for operation of the diesel engine exposure systems. This work was performed in conjunction with the NERC, at the Lovelace Respiratory Research Institute, with funds from multiple government and industry sponsors, including the US EPA. This work was also funded in part by a Pilot Project Award from the NIEHS Development Center Grant (P20-ES09781-04) (K.S.H.); the Health Effects Institute, an organization jointly funded by the US EPA, and automotive manufacturers (K.S.H); and the National Institutes of Health grant HL-66964 (K.S.H.). Lovelace Respiratory Research Institute is fully accredited by the Association for the Assessment and Accreditation of Laboratory Animal Care International.
Hashiguchi	2015	Japan	This study was supported by a Health and Labour Sciences Research Grant (H24-kagakushitei-009) from the Ministry of Health, Labour and Welfare, Japan and partly by grant-in-Aid for Science Research (No. 26460183) from the Japan Society for the Promotion of Science.
Hirota	2015	Canada	The work was funded by the Canadian Institutes for Health Research.
Hobson and Everard	2007	UK	Green fluorescent protein-expressing laboratory A2 strain of RSV (rg-RSV) and red fluorescent protein-expressing B strain (rr-RSV) were kindly donated by Dr M. Peeples. Lynsey Hobson was supported by the Sheffield Children’s Hospital Research fund.
Ivanciuc	2019	USA	This work was partially supported by NIH grants ES026782 (RPG), AI125434 (AC), ES006676 (AC, NIEHS), AI062885 (RPG and AC).
Kaan and Hegele	2003	Canada	This work was supported by The Canadian Institutes of Health Research and The British Columbia Lung Association. The authors thank Dr. Renaud Vincent, Environmental Health Canada, for providing PM10 particles; Mr. Stuart Greene and Mr. Dean English for expert photography assistance and Ms. Lynne Carter for assistance in handling of experimental animals; and Dr. Stephan van Eeden for constructive

			discussion. Dr. Robert Alain, INRS-Institut Armand-Frappier, (Laval, QC, Canada) performed negative staining transmission electron microscopy of RSV stocks.
Lambert 2003 " Effect of Preexposure to Ultrafine Carbon Black…"	2003	USA	This work was supported in part by a National Research Service Award from the National Institute of Environmental Health Sciences.
Lambert 2003	2003	USA	no funding
Mebratu	2016	USA	no funding
Modestou	2010	USA	This work was supported by Public Health Service grants HL082505 and HL075559 from the National Heart, Lung, and Blood Institute. The University of Iowa Cell Culture Core Repository is supported by grants from the National Institutes of Health and Cystic Fibrosis Foundation.
Phaybouth	2006	USA	This work was supported, in part, by National Institute of Environmental Health Sciences Grant P30 ES-012072 and by the Tobacco Master Settlement through a cooperative research agreement with the University of New Mexico.
Poon	2019	USA	This work was supported by grants made available to PG (Flight Attendant Medical Research Institute YCSA113380 and CIA160005 and the Alpha-1 Foundation 493373) and to MS (Flight Attendant Medical Research Institute, CIA160011and CIA13033, as well as James & Esther King Biomedical Program of the State of Florida, #5JK02).
Raza	1999	UK	This work was supported by the Chest, Heart and Stroke Association, Scotland.
Smallcombe	2020	USA	This work was supported by the Mark Lauer Pediatric Research Grant, Cleveland Clinic 610 Children's (CCS/FR); as well as National Institutes of Health grants K08-AI-112781(FR),

			R01-HL-148057 (FR), and R01-HL-061007 (GP). This work also utilized the Leica SP8 confocal microscope that was purchased with funding from NIH SIG Grant S10-OD019972.
Soukup, Koren, Becker	1993	USA	no funding
Urrego	2009	USA	This research was supported in part by grants from the National Institutes of Health (NHLBI HL-61007 and NICHD NCS-07-11) to Dr. Giovanni Piedimonte.

Table S4. Funding sources of the studies included in the review.
